# PEGylated Bilirubin-coated Iron Oxide Nanoparticles as a Biosensor for Magnetic Relaxation Switching-based ROS Detection in Whole Blood

**DOI:** 10.7150/thno.39662

**Published:** 2020-01-12

**Authors:** Dong Yun Lee, Sukmo Kang, Yonghyun Lee, Jin Yong Kim, Dohyun Yoo, Wonsik Jung, Soyoung Lee, Yong Yeon Jeong, Kwangyeol Lee, Sangyong Jon

**Affiliations:** 1 Graduate School of Medical Science and Engineering, Korea Advanced Institute of Science and Technology (KAIST), 291 Daehak-ro, Daejeon 34141, Republic of Korea; 2 Department of Nuclear Medicine, Asan Medical Center, University of Ulsan College of Medicine, 88, OLYMPIC-RO 43-GIL, Seoul 05505, Republic of Korea; 3 Department of Biological Sciences, KAIST, 291 Daehak-ro, Daejeon 34141, Republic of Korea; 4 KAIST Institute for BioCentury, KAIST, 291 Daehak-ro, Daejeon 34141, Republic of Korea; 5 Center for Precision Bio-Nanomedicine, KAIST, 291 Daehak-ro, Daejeon 34141, Republic of Korea; 6 Department of Radiology, Chonnam National University Hwasun Hospital, 322 Seoyang-ro, Hwasun 58128, Republic of Korea; 7 Department of Chemistry, Korea University, 145 Anam-ro, Seoul 02841, Republic of Korea

**Keywords:** Bilirubin nanoparticles, Biosensors, Iron oxide nanoparticles, Magnetic relaxation switching, Reactive oxygen species

## Abstract

**Rationale**: Magnetic relaxation switching (MRSw) induced by target-triggered aggregation or dissociation of superparamagnetic iron oxide nanoparticles (SPIONs) have been utilized for detection of diverse biomarkers. However, an MRSw-based biosensor for reactive oxygen species (ROS) has never been documented.

**Methods**: To this end, we constructed a biosensor for ROS detection based on PEGylated bilirubin (PEG-BR)-coated SPIONs (PEG-BR@SPIONs) that were prepared by simple sonication via ligand exchange. In addition, near infra-red (NIR) fluorescent dye was loaded onto PEG-BR@SPIONs as a secondary option for fluorescence-based ROS detection.

Resul**ts**: PEG-BR@SPIONs showed high colloidal stability under physiological conditions, but upon exposure to the model ROS, NaOCl, *in vitro*, they aggregated, causing a decrease in signal intensity in T2-weighted MR images. Furthermore, ROS-responsive PEG-BR@SPIONs were taken up by lipopolysaccharide (LPS)-activated macrophages to a much greater extent than ROS-unresponsive control nanoparticles (PEG-DSPE@SPIONs). In a sepsis-mimetic clinical setting, PEG-BR@SPIONs were able to directly detect the concentrations of ROS in whole blood samples through a clear change in T2 MR signals and a 'turn-on' signal of fluorescence.

**Conclusions**: These findings suggest that PEG-BR@SPIONs have the potential as a new type of dual mode (MRSw-based and fluorescence-based) biosensors for ROS detection and could be used to diagnose many diseases associated with ROS overproduction.

## Introduction

Superparamagnetic iron oxide nanoparticles (SPIONs) have been used extensively for diverse biomedical applications, including as T2 contrast agents for magnetic resonance imaging (MRI), drug-delivery carriers, heat-generating materials in magnetic hyperthermia, and biosensors based on the principle of “magnetic relaxation switching (MRSw)” 1,2. Among these applications, MRSw-based assays have shown the potential for use as *in vitro* diagnosis and biosensing systems for variable biomarkers, including oligonucleotides, proteins and even small molecules 3. A very recent study has demonstrated the successful use of aggregation-induced MRSw-based imaging of enzymatic activity and glutathione in the tumor microenvironment in an *in vivo* setting 4,5. However, few MRSw-based probes that can detect pathophysiological changes, such as pH and reactive oxygen species (ROS)-mediated oxidative stress under certain disease conditions, have developed. Although a number of fluorescence-based probes have reported for ROS detection 6-8, auto-fluorescence of endogenous fluorescent species in medical samples of blood and serum strongly interfere with such fluorescence-based signals, leading to poor signal-to-noise ratio and reliability.

In particular, ROS play an important role in the pathogenesis and progression of inflammatory diseases and cancers 9; thus, high levels of ROS are found in patients with these diseases. Especially, excess amount of ROS generation on the onset of sepsis has been suggested as an early biomarker. Currently available biosensing systems for detection of such aberrant oxidative stress are confined to probe indirect surrogate markers such as ROS-induced by-products, chloramine and malondialdehyde 10,11, highlighting the need of a biosensor that enables direct ROS detection in whole blood.

**Figure 1:**
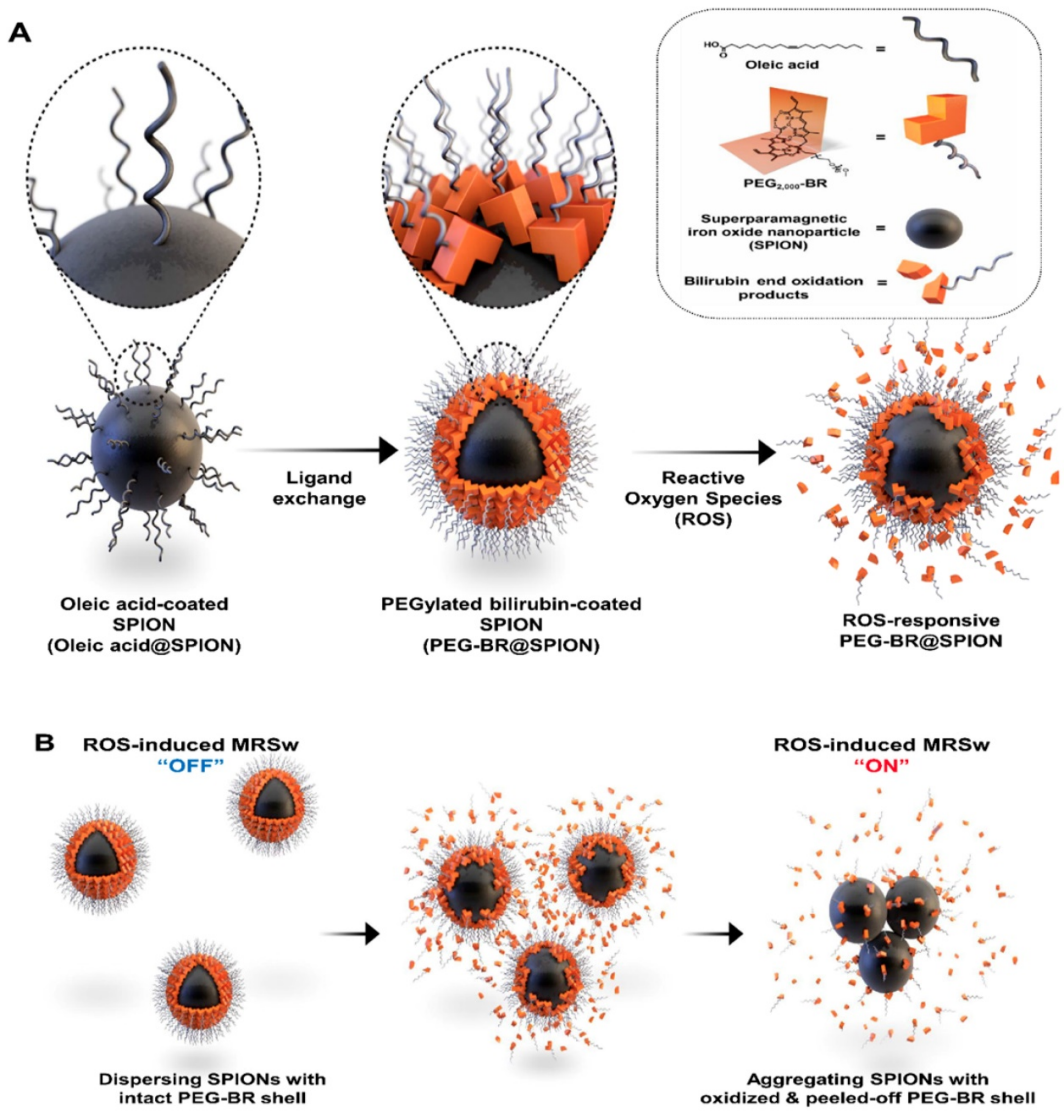
** A synthetic scheme and mode of action of PEGylated-bilirubin coated superparamagnetic iron oxide nanoparticles (PEG-BR@SPIONs).** (A) Synthetic scheme for fabricating ROS-responsive PEG-BR@SPIONs from oleic acid-coated SPIONs by ligand exchange. (B) Proposed mechanisms illustrating how PEG-BR@SPIONs respond to ROS stimuli and how the resulting responsiveness facilitates real-time ROS monitoring using a MR-based tactic.

We have recently reported a number of potential biomedical applications of bilirubin-based nanoparticles (BRNPs), which show potent anti-inflammatory efficacy in various animal models and have the potential for use as dual stimulus (ROS and light)-responsive drug-delivery carriers for cancer therapy 12-18. These BRNPs, which are prepared by self-assembly of poly(ethylene glycol) (PEG)-modified bilirubin (PEG-BR), show a switch in water solubility from hydrophobic to hydrophilic as well as degradation of the BR core into fragments upon oxidation by various ROS and consequently undergo disassembly of the nanoparticles 13. Furthermore, exploiting the fact that bilirubin can function as an endogenous chelator of various metal ions in the body 19, we successfully constructed cisplatin-chelated BRNPs that could be used as a photoacoustic and photothermal agent for combined cancer imaging and therapy 20. Encouraged by this metal-chelating power and ROS-responsiveness, we hypothesized that PEG-BR could also be used to coat the surface of SPIONs through metal coordination, producing PEG-BR-coated SPIONs, designated PEG-BR@SPIONs (Figure **1A**). Upon exposure to ROS, the PEG-BR coating layer would undergo oxidation and be converted to water soluble PEG-biliverdin, which would become further oxidized to BR oxidation end products (fragmentized BR) and be easily peeled off from the core SPIONs 21, which, in turn, start aggregating with one another in the biological milieu owing to attractive forces and instability, resulting in a change in the MR signal for potential MRSw-based biosensor (Figure **1B**).

## Results & Discussion

### Synthesis and *in situ* ROS-responsiveness of the PEG-BR@SPIONs

**Figure 2:**
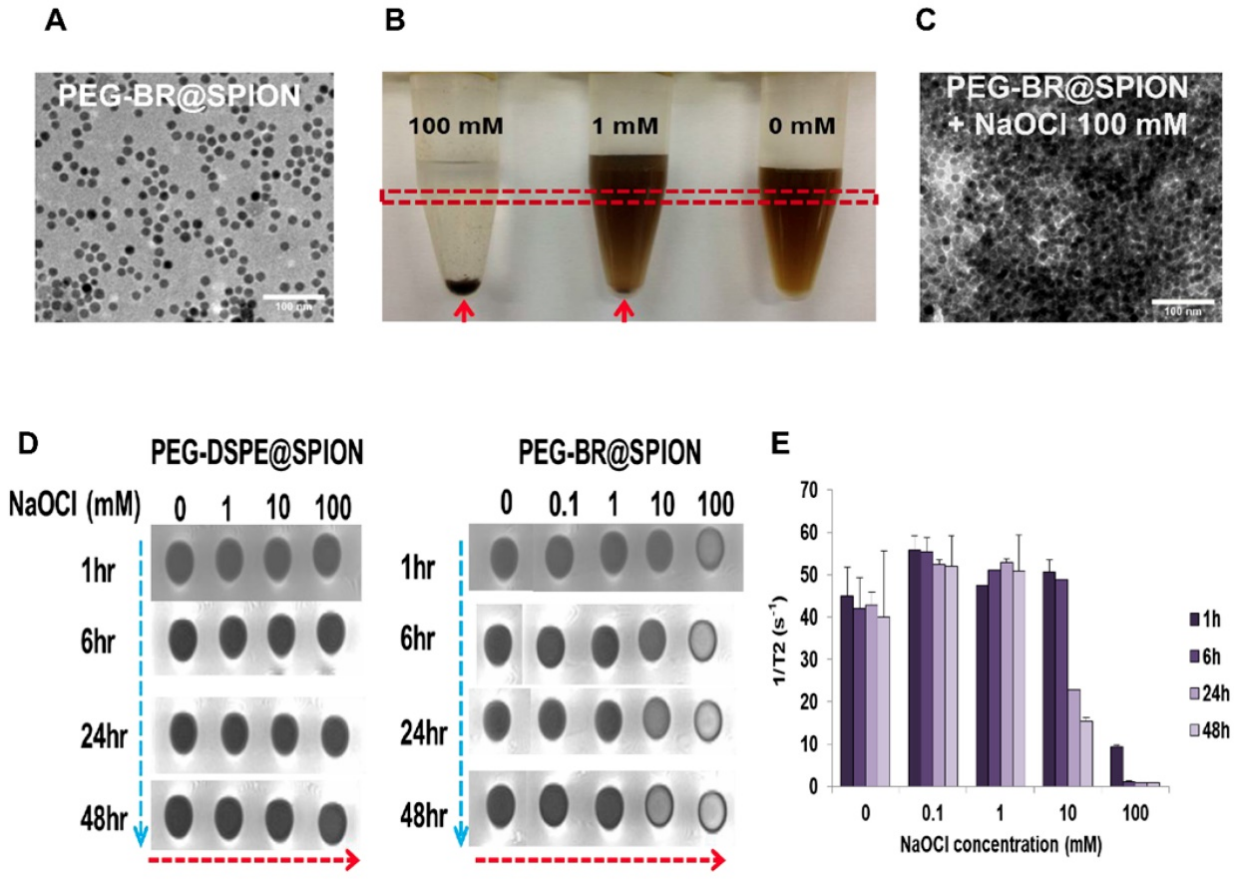
***In situ* ROS-responsiveness of the PEG-BR@SPIONs.** (A) TEM image of as-prepared PEG-BR@SPIONs. (B) Photograph of PEG-BR@SPION (Fe, 10 mM) solutions after reacting with different concentrations of NaOCl. Red arrow indicates precipitate of aggregated SPIONs. (C) TEM image of as-prepared PEG-BR@SPIONs after ROS treatment. (D) Phantom images showing time- and ROS concentration-dependent T2 MR contrast changes for each indicated SPION. (E) A bar graph for T2 values calculated from phantom images of PEG-BR@SPIONs as a function of different concentrations of NaOCl at each time point.

PEG-BR@SPIONs were prepared by simple sonication-based mixing of oleic acid-stabilized SPIONs in hexane with an aqueous solution of PEG-BR via ligand exchange 22. This process is favorable because BR, owing to its unique 'ridge-tile' shape 23, is able to coordinate at surface of the nanoparticles using two available amide and carboxylic acid functional groups present in both sides of BR as illustrated in Figure **1A**, having a higher affinity for SPIONs than oleic acid 24. A representative transmission electron microscopy (TEM) image of as-prepared PEG-BR@SPIONs shows uniformly distributed iron oxide nanoparticles with an average diameter of ~15 nm (Figure **2A**). The amount of PEG-BR coating in the nanoparticles was found to be ~5.75 ± 1.06 μg PEG-BR/mM Fe, determined using UV-Vis spectroscopy and inductively coupled plasma mass spectroscopy (ICP-MS), as described in the Experimental Section. As a control group that does not respond to ROS, SPIONs coated with the PEGylated phospholipid, PEG2000-DSPE (1,2-distearoyl-sn-glycero-3-phosphoethanolamine-N-[amino(polyethylene glycol) 2000]) (PEG-DSPE@SPIONs), were prepared using the same basic sonication-based mixing process as that used to prepare PEG-BR@SPIONs. The two SPIONs exhibited distinct colors in aqueous solution and spectral patterns in Fourier-transform infrared (FT-IR) spectra (Figures **S1** and **S2**), owing to differences in their coating layers. Next, the colloidal stability of PEG-BR@SPIONs was tested for 5 days under various conditions, including in distilled water (DW), cell culture medium, and mouse plasma (Figure **S3**). Although there was some variability, the average hydrodynamic size of nanoparticles remained overtly steady at approximately 30-50 nm over the course of these measurements. The transverse relaxivity (*r*2 =1/T2) of PEG-BR@SPIONs, determined from MR phantom images as a function of concentrations, was calculated to be 146.12 mM^-1^s^-1^, which is higher than that of the clinically approved contrast agent, Feridex (108.2 mM^-1^s^-1^) (Figure **S4**) 25. We next examined the responsiveness of PEG-BR@SPIONs to ROS. Sodium hypochlorite (NaOCl) was used as a ROS source, because it generates ROS typical of those produced by phagocytes during bacterial infection 26. ROS induced a distinct, concentration-dependent change in the colloidal stability of PEG-BR@SPIONs. Whereas PEG-BR@SPIONs were in a well-dispersed colloidal state in the absence of ROS, the highest concentration of NaOCl (100 mM) immediately led to complete precipitation of the nanoparticles (Figure **2B**). Such ROS-responsive aggregation phenomena were further confirmed by TEM (Figure **2C**) and transmission light microscopy (Figure **S5**). It should be further noted that the MR signal of PEG-BR@SPIONs became gradually attenuated at the region of interest (ROI; indicated by the red box in Figure **2B**) in T2-weighted phantom images with increases in ROS concentration and incubation time (Figure **2D** and** 2E**), whereas PEG-DSPE@SPIONs signals were largely unchanged, indicative of a lack of ROS responsiveness by the latter nanoparticles. The difference in ROS responsiveness between the two nanoparticle systems was also clearly evident by a change in the color of the solution upon exposure to NaOCl-derived ROS (Figure **S6**). UV-Vis spectroscopic measurements further confirmed the clear difference in ROS reactivity between PEG-BR@SPIONs and PEG-DSPE@SPIONs (Figures **S7** and **S8**). For PEG-BR@SPIONs, the change in the absorbance at 420 nm indicates that the chemical structure of BR is modified (or fragmentized) upon reaction with ROS. A similar tendency for PEG-BR@SPIONs to undergo ROS-induced aggregation was observed for an azo radical generator, 2,2'-azobis(2-amidinopropane) dihydrochloride (AAPH) (Figure **S9)**. Relative responsiveness or sensitivity of PEG-BR@SPIONs toward various ROS was assessed. PEG-BR@SPIONs showed much greater reactivity toward both NaOCl and potassium superoxide (KO_2_) compared to AAPH and H_2_O_2_ (Figure **S10**). Interestingly, PEG-BR@SPIONs showed no appreciable visual change in the presence of H_2_O_2_, even after treating for 24 hours, suggesting differential reactivity of PEG-BR@SPIONs to the type of ROS. These results suggest that PEG-BR@SPIONs have the potential for use as MR-based biosensors for the detection of certain types of ROS.

### *In vitro* ROS-responsiveness of the PEG-BR@SPIONs

**Figure 3:**
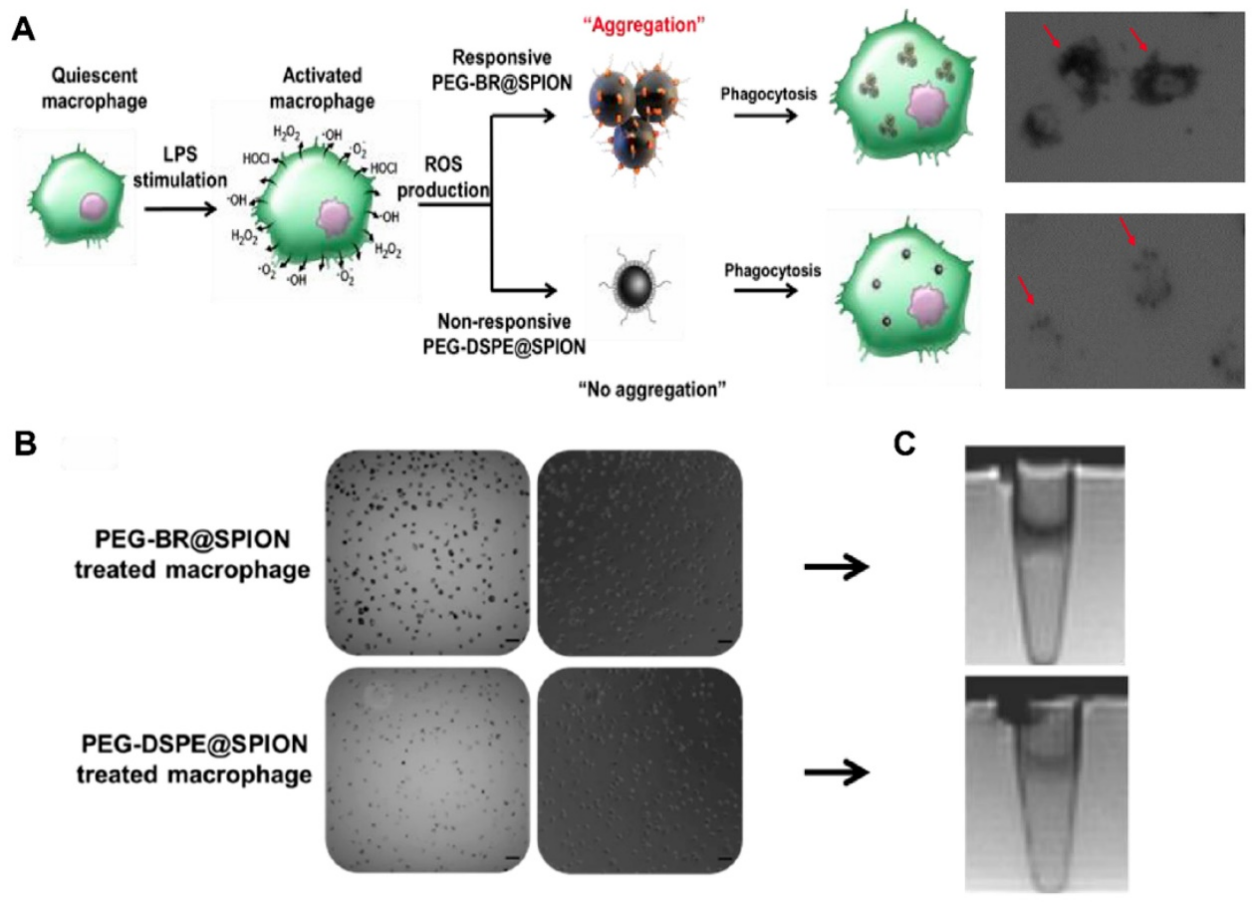
***In vitro* ROS-responsiveness of the PEG-BR@SPIONs.** (A) Schematic figure with corresponding enlarged images of cell uptake showing differences in intracellular uptake of two types of SPIONs by LPS-stimulated macrophages. (B) Bright-field and phase-contrast images of RAW 264.7 cells 6 hours after treatment with LPS and each SPION (Scale bar: 50 µm). (C) Corresponding T2 MR phantom images of RAW 264.7 cells (n = 3).

Next, we examined whether PEG-BR@SPIONs could respond to ROS in an actual physiological setting. It is known that, under certain pathological conditions such as infection, macrophages produce a considerable amount of ROS 27. To mimic this situation, we induced RAW 264.7 mouse macrophage cells to overproduce various ROS in the culture medium by stimulating them with lipopolysaccharide (LPS) and then incubated them with PEG-BR@SPIONs or PEG-DSPE@SPIONs (Figure **3A**). We first examined ROS levels in the culture medium and in cells. LPS treatment induced a considerable increase in ROS production both in the culture medium (Figure **S11**) and inside the cells (Figure **S12**). As seen in bright field images (Figure** 3B**), LPS-stimulated macrophages treated with PEG-BR@SPIONs were much darker in color, indicating greater cellular uptake of these SPIONs than PEG-DSPE@SPIONs. The results of Prussian blue staining assays suggested that PEG-BR@SPIONs were mainly trapped in the cytoplasm of cells (Figure **S13**). This clear difference in cellular uptake between the two types of nanoparticles was also confirmed in an MRI cell phantom study, which showed that T2-weighted MR images of PEG-BR@SPIONs were much darker than those of PEG-DSPE@SPIONs (Figure **3C**). However, there was no difference in mRNA expression levels of ROS-producing enzymes in cells treated with either type of nanoparticle (Figure **S14**). We speculated that the overproduced ROS from the LPS-activated macrophages caused aggregation of SPIONs after peeling off from the PEG-BR shell, allowing the resulting aggregates to be taken up by cells with higher efficiency than the individually dispersed PEG-DSPE@SPIONs.

### Magnetic Relaxation Switching-based ROS Detection of PEG-BR@SPIONs in whole blood

**Figure 4:**
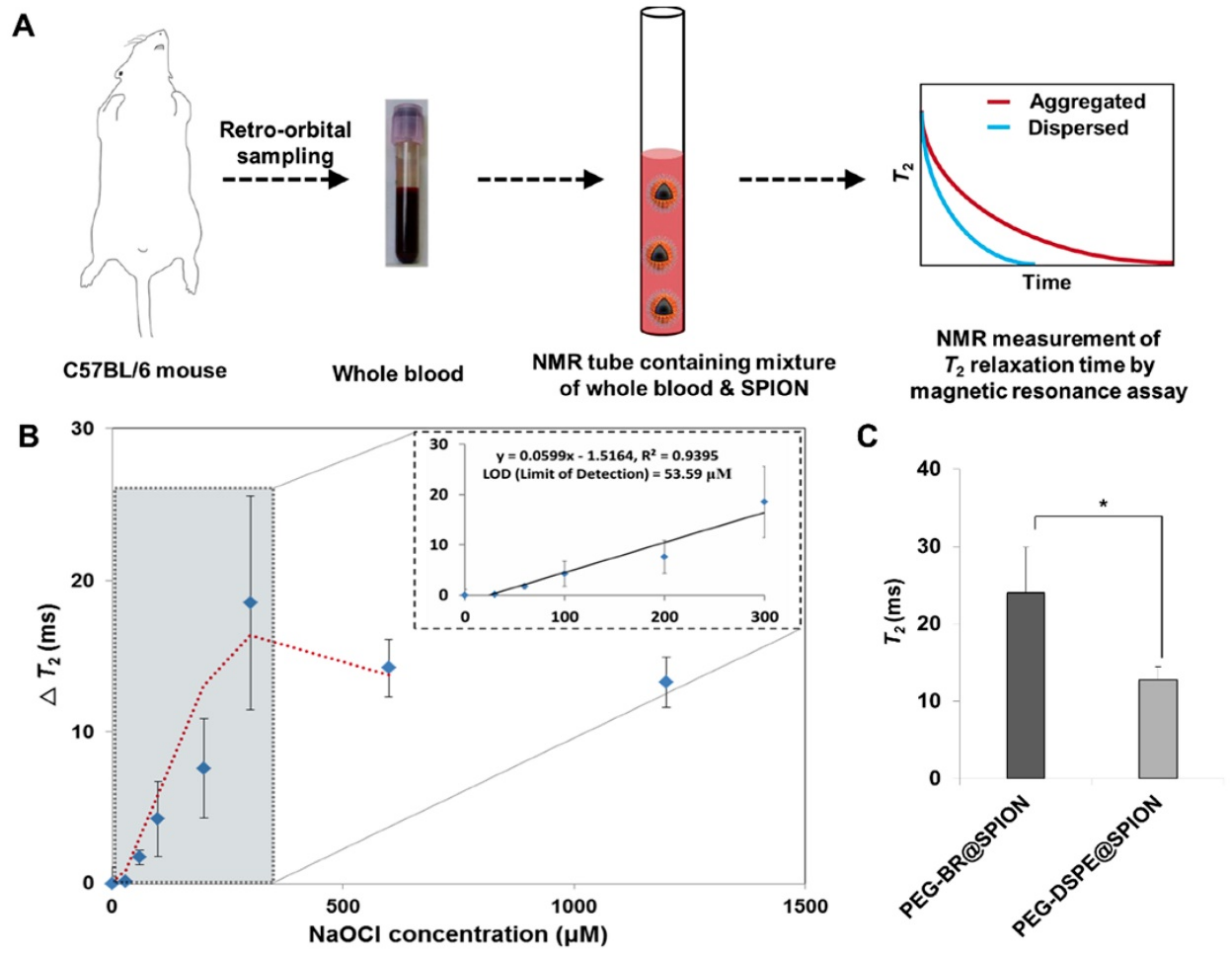
** Magnetic Relaxation Switching-based ROS Detection of PEG-BR@SPIONs in whole blood.** (A) Basic scheme and concept of the whole-blood assay for MRSw-based ROS detection using PEG-BR@SPIONs. (B) ΔT2 as a function of NaOCl concentration 12 hours after sample mixing (n = 4). Inset: linear detection range. (C) Comparison of T2 between the two assay groups in LPS-treated peritonitis model (n = 4~5 mice/group; *P < 0.05).

Unlike optical and electrochemical biosensors 28, MR-based sensors are largely unaffected by interference from signals of intrinsic biological species or the turbidity of biological samples, such as blood 29-30. By assessing the genuine ROS level under MRSw in real biological milieu, like whole blood, we would be able to evaluate the severity of sepsis and potentially select the patients with sepsis who might be beneficial from antioxidant therapy 31. Accordingly, we examined the feasibility of using PEG-BR@SPIONs as an MRSw-based biosensor for ROS detection by incubating them in whole blood of mice spiked with known concentrations of NaOCl (Figure **4A**). Upon exposure to NaOCl (300 μM) in whole blood, PEG-BR@SPIONs showed a gradual increase in ΔT2 (change in T2 relaxation time) (Figure **S15**), corresponding to 'Type 2 MRSw' behavior of ~15 nm SPIONs 32-33. Further experiments revealed that PEG-BR@SPIONs had a limit of detection (LOD) for NaOCl in blood of 53.6 μM and their response plateaued at greater than 300 μM NaOCl (Figure **4B**). It is well known that oxidative stress is significantly elevated during bacterial infection-induced sepsis 34. We thus next applied the MRSw-based biosensor to ROS detection in a disease setting using LPS-induced peritonitis in mice as a sepsis model. Mouse temperature, monitored using a rectal probe, was significantly reduced 6 hours after LPS injection (Figure **S16**). In addition, the number of white blood cells and platelets decreased in the LPS-induced sepsis mouse and the differential count of neutrophils among white blood cell subtypes appreciably increased (Figure **S17**), satisfying the diagnostic criteria of sepsis 35. After incubating in the blood drawn from sepsis-model mice, control PEG-DSEP@SPIONs showed only a modest increase in T2, whereas PEG-BR@SPIONs showed a significantly greater increase in T2 (12.79 ms ± 1.63 vs. 24.03 ms ± 5.85, p < 0.05) (Figure **4C**), indicating the potential of the latter SPION to act as a biosensor for ROS detection in a clinical setting.

### Fluorescence-based ROS-responsiveness in NIR dye loaded PEG-BR@SPIONs

**Figure 5:**
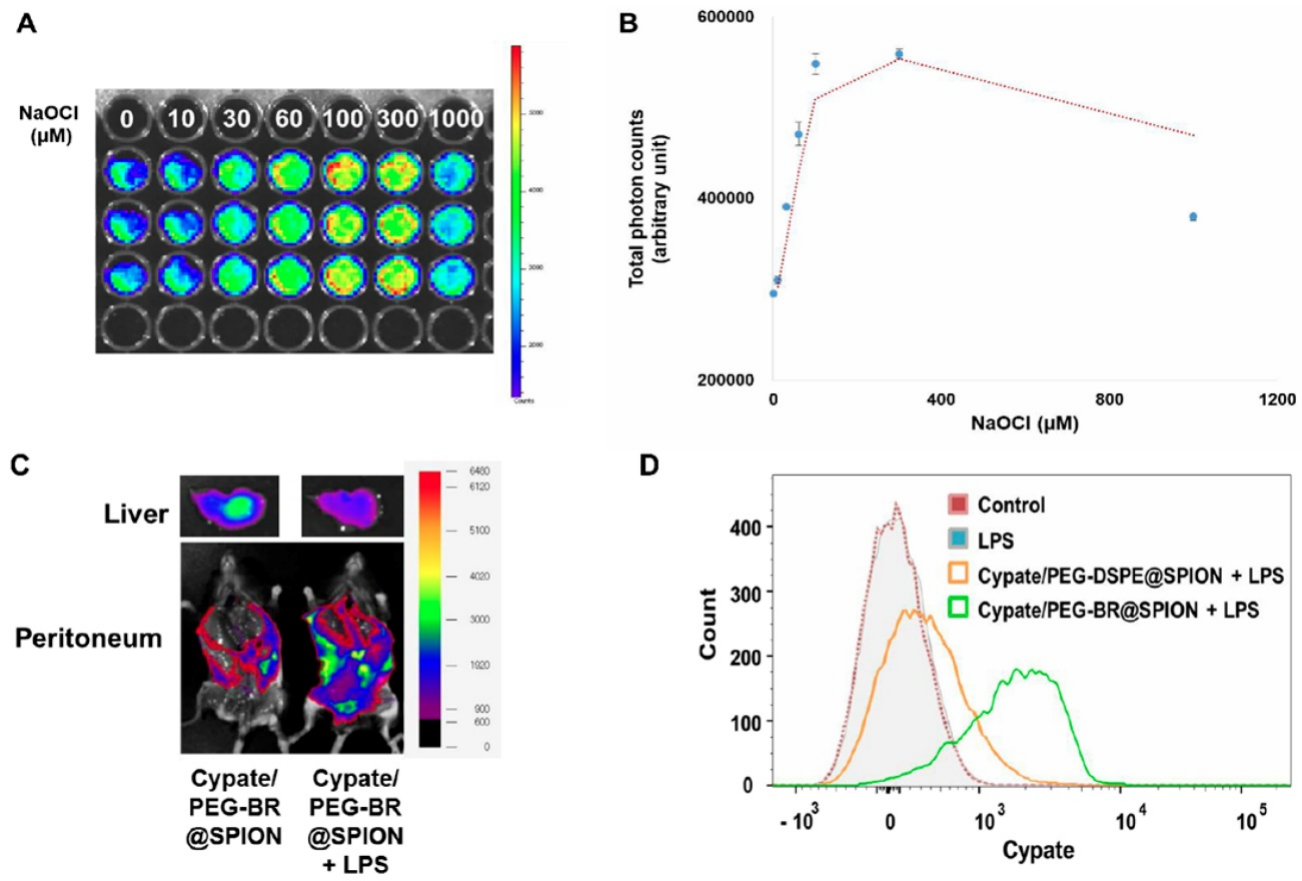
** Fluorescence-based ROS Detection in NIR dye loaded PEG-BR@SPIONs.** (A) *In si*tu fluorescence images and (B) corresponding photon counts of the whole blood containing Cypate/PEG-BR@SPIONs as a function of NaOCl concentrations 3 hours after sample mixing (n = 3). (C) *Ex vivo* fluorescence images of Cypate/PEG-BR@SPIONs around the abdominal wall and in the liver, with and without intraperitoneal LPS treatment. (D) Representative flow cytometry histogram of resident peritoneal cells treated as indicated.

Encouraged by the successful demonstration of MRSw-based ROS detection in a clinical-mimetic setting, we next explored the possibility of extending the application of PEG-BR@SPIONs to fluorescence-based ROS detection. As a proof-of-concept study, we loaded the hydrophobic near-infrared (NIR) dye, cypate 36, into each nanoparticle system, generating Cypate/PEG-BR@SPIONs and Cypate/PEG-DSPE@SPIONs. The fluorescence of cypate became substantially quenched after being loaded into PEG-BR@SPIONs, presumably owing to electronic interactions between cypate and BR; however, treatment of detergent Triton X-100 (0.5%) led to the full recovery of fluorescence (Figure **S18**), suggesting release of the dye from shell of nanoparticles. This 'off' to 'on' signaling switch of Cypate/PEG-BR@SPIONs was applied to fluorescence-based detection of ROS by incubating them in whole blood of mice spiked with known concentrations of NaOCl. As shown in Figure **5A** and **5B**, the fluorescence signal intensity increased in proportion to the ROS concentrations in the blood as high as 300 μM with LOD of 31.49 μM, indicating release of the dye from the nanoparticles upon peeling off the PEG-BR coating layer. At high concentration of ROS (1 mM), however, the fluorescence signal became rather dropped, presumably due to oxidation of the dye itself, implying existence of the optimal range of ROS detection. For *in vivo* demonstration, LPS-induced peritonitis model was established with injecting these SPIONs into the peritoneal cavity, where activated macrophages had infiltrated in response to local inflammation 37. Six hours after injection, mice were sacrificed, laparotomized, and imaged by *in vivo* fluorescence imaging. As shown in Figures **5C** and** S19**, LPS-injected, mice showed a much higher signal along the abdominal wall, and a much lower signal in the liver than untreated mice, suggesting substantial release of cypate from nanoparticles in the inflamed peritoneum as a result of ROS-mediated detachment from PEG-BR shell. Prussian blue staining of the excised abdominal wall from LPS-injected mice showed a linear, spotty distribution of SPIONs along the parietal peritoneum, confirming the retention of nanoparticles in the inflamed site (Figure **S20**). Using a peritoneal lavage method, we next extracted resident macrophages directly from the peritoneum of LPS-injected mice treated with either Cypate/PEG-BR@SPIONs or Cypate/PEG-DSPE@SPIONs, and analyzed the cypate fluorescence-positive cell population by flow cytometry. Whereas mice injected with Cypate/PEG-DSPE@SPIONs showed an increase in the fluorescence-positive cell population compared with control mice, this population was dramatically increased in LPS-injected mice that received ROS-responsive Cypate/PEG-BR@SPIONs (Figure **5D**), suggesting increased uptake of detached cypate by activated macrophages in the inflamed peritoneum. Mean fluorescence intensity values also showed a clear difference between two groups (Figure **S21**). Taken together, these results clearly suggest that PEG-BR@SPIONs can be constructed as a dual mode (MRSw/fluorescence) biosensor for ROS detection.

## Conclusion

In this study, we developed a SPION-based biosensor for ROS detection that can function in a physiological milieu. The intrinsic metal-chelating ability of bilirubin in PEG-BR enabled it to stably coat SPIONs, and its ROS-responsiveness enabled it to act as a biosensor for ROS. This is a first report of an MRSw-based biosensor that is able to detect various ROS in blood without interference. Therefore, we anticipate that the PEG-BR-coated SPIONs developed herein could be used to detect elevated ROS in pathologic tissues by MRI and identify the ROS-enriched status associated with the onset of acute inflammatory diseases in an MRSw-based whole-blood assay.

## Methods

### Materials

Bilirubin (BR) was purchased from Tokyo Chemical Industry Co., Ltd. (Tokyo, Japan), and polyethylene glycol(2000)-DSPE (ammonium salt) (PEG2000-DSPE) was purchased from Avanti Polar Lipids (Alabaster, AL, USA). EDC [1-ethyl-3-(3-dimethylaminopropyl)carbodiimide], mPEG2000-NH2, sodium hypochlorite (NaOCl), hydrogen peroxide (H2O2), 2,2'-azobis(2-methylpropionamidine) dihydrochloride (AAPH), gelatin from porcine skin, Prussian blue soluble and neutral red were all purchased from Sigma Chemical Co., Inc. (St. Louis, MO, USA).

### Synthesis of iron oxide nanoparticles

The superparamagnetic Fe3O4 MNPs were prepared by recrystallization of hematite, as described by Cheng et al 38. A mixture of bulk α-Fe2O3 powder (3 mM) and oleic acid (26 mM, 9.0 ml) was placed in a three-necked, round-bottom flask and heated to 320 °C at ~15 °C/min. The temperature was controlled with a heating mantle and a thermocouple. During the reaction, the mixture bubbled and spilled owing to the generation of water, an effect that was reduced by distillation. During the reaction, the color of the reactant changed from red to brown and then to black. The resulting products were diluted with hexane, and then centrifuged at 5000 rpm. The resulting pellets were washed with ethanol, dried, and weighed. The supernatant was precipitated with a copious amount of acetone, and the precipitate was washed with acetone and ethanol two or three times. The precipitated NPs were redispersed in hexane.

### Synthesis of PEGylated BR

All reactions involving BR were performed under dim light. PEG-BR and BRNPs were synthesized and formulated according to a previously reported method, with slight modifications 12. In brief, 0.5 mM (ZZ)-BR-IX-alpha was dissolved in 5 ml of dimethyl sulfoxide (DMSO), to which 150 μl trimethylamine (TEA) was added. After BR had completely dissolved, 0.6 mM EDC was added and the mixture was stirred for 10 minutes at room temperature (RT) to activate BR. Then, 0.2 mM mPEG_2000_-NH_2_ was added to the mixture, and the reaction was allowed to proceed with stirring for 4 hours at RT under a nitrogen atmosphere. Free BR was removed by adding methanol to the reaction mixture and centrifuging the solution at 2,000 × g for 10 minutes. The supernatant was harvested and evaporated, followed by the addition of ether. The resulting precipitate was dissolved in chloroform and purified by silica column chromatography using chloroform/methanol as the mobile phase, after which solvents were evaporated to yield PEG-BR powder.

### Preparation of single iron oxide nanoparticles coated with PEGylated BR

Iron oxide nanoparticles (30 mg) were dissolved in 10 ml of hexane. Small aliquots (200 μl) of iron oxide nanoparticles suspended in hexane solutions were layered on a 2-ml aqueous solution of PEG-BR (250 μM), after which the oil-in-water mixture was sonicated for 15 minutes using an ultrasonic processor. The resulting emulsion was stirred under dim light for 6 hours to evaporate hexane, and centrifuged at 5,000 rpm for 10 minutes to remove any aggregates. The supernatant was then filtered through nylon membrane filters with a pore size of 0.2 μm (Whatman, GE Healthcare Life Sciences, UK), and purified using a rare earth magnet to extract PEG-BR-coated iron oxide nanoparticles. The fabrication of PEG-DSPE-coated iron oxide nanoparticles followed a similar procedure, but cypate loading was accomplished using a slight modification of the above method. Very small aliquots (10 μl) of cypate dissolved in chloroform solution were layered on a 2-ml aqueous solution of PEG-BR immediately after layering the hexane solution of iron oxide nanoparticles. The resulting oil-in-water solution was then sonicated and the subsequent steps were repeated as described above.

### Characterization of iron oxide nanoparticles

The hydrodynamic size distribution of iron oxide nanoparticles was evaluated using a Nanosizer ZS 90 (Malvern Instruments, Ltd., Malvern, UK), and their gross morphology was characterized by transmission electron microscopy (TEM; Tecnai G2 F30, Eindhoven, Netherlands). UV-Vis spectra were acquired using a NEOSYS2000 system (Scinco, Twin Lakes, WI, USA), and Fourier transform infrared (FT-IR) spectra were acquired using a Nicolet iS50 system (Thermo Fisher Scientific, Inc., Waltham, MA, USA). Iron was quantified by inductively coupled plasma mass spectroscopy (ICP-MS) using an Elan DRC II system (Concord, Ontario, Canada).

### *In situ* test of ROS responsiveness

ROS-induced gross color or absorbance changes were observed by mixing iron oxide nanoparticles (Fe, 10 mM) with an equal volume of solutions containing different concentrations (0.1, 1, 10, 100 mM) of four types of ROS (AAPH, NaOCl, H_2_O_2_, KO_2_) in a microcentrifuge tube and incubating under dim light for up to 48 hours at RT. Microscopic changes were tracked by placing 100 μl aliquots of as-prepared solutions in a 96-well plate and observing under a light microscope. TEM images were obtained before and after treatment with NaOCl, prepared as described above and used as a representative ROS. For fluorescence imaging, whole blood drawn from mice were used and incubated with the cypate-loaded PEG-BR@SPION (Fe: 1 mM).

### Cell lines and animals

The mouse macrophage cell line RAW 264.7 was obtained from the Korean Cell Line Bank (Seoul, Korea) and cultured in recommended media (DMEM; Welgene, Daegu, South Korea) supplemented with 10% fetal bovine serum (FBS) and 1% penicillin/streptomycin at 37°C in a humidified 5% CO2 atmosphere. Female wild-type C57BL/6 mice (6-8 weeks old) were purchased from Orient Bio, Inc. (Seongnam, Korea) and housed under pathogen-free conditions in an animal facility at the Korea Advanced Institute of Science and Technology. All animal procedures were carried out in accordance with ethical and scientific care procedures approved by the Korea Advanced Institute of Science and Technology Institutional Animal Care and Use Committee (KAIST-IACUC; approval number: KA2015-09).

### MRI imaging

Microcentrifuge tubes were used for *in situ* and *in vitro* MR imaging. All MR imaging was performed using a 3.0 T clinical MRI scanner (MAGNETOM Tim Trio; Siemens Medical Solutions, Erlangen, Germany). T2 relaxation time and T2 relaxivity coefficients were measured from images obtained with the following parameters: TR (repetition time) = 1950 ms; TE (echo time) = 14, 28, 41, 55, 69, 83, 97, 110, 124, 138, 152, 167, 179, 193, 207, and 220 ms. Iron oxide nanoparticle uptake by RAW 264.7 cells was visualized using a cell phantom study in which a layer of 10% gelatin (200 μl) was first molded into the bottom of a tube. RAW 264.7 cells (5 × 10^6^ cells) were fixed by suspending in 50 μl of 4% paraformaldehyde and embedded in the middle layer of the tube with the same volume of 10% gelatin, which was then covered with 150 μl of cell-free gelatin. Coronal T2-weighted MR images was acquired with the following parameters: TR/TE, 3,000 ms/83 ms; flip angle, 150°; field of view, 150 × 150 mm; slice thickness, 2 mm; matrix number, 448 × 314. For the analysis of relaxation time, circular regions of interest (7 mm in diameter) were placed in the center of phantom tubes; areas with air-susceptibility artifacts were excluded. The signal intensity (SI) of T2-weighted MR images was obtained by averaging the middle five regions of interest in the phantom volume.

### *In vitro* test of ROS responsiveness

RAW 264.7 cells (5 × 10^3^ cells/well) were seeded in 96-well plates and incubated for 24 hours. Fresh medium containing LPS (5 μg/ml) was then added and cells were co-incubated with each SPION (Fe, 1 mM) for 6 hours. After washing, cells were imaged by bright-field and phase-contrast microscopy using an ImageXpress Micro XL High-Content Imaging System (Molecular Devices, Sunnyvale, CA, USA).

### Measurement of ROS production

Extracellular ROS production was determined by measuring ROS released into the medium using an Acridan Lumigen PS-3 assay 39. RAW 264.7 cells (5 × 10^3^ cells/well) were seeded in clear-bottom, 96-well black plates and treated with different concentrations of LPS (0, 1, 5, 10 μg/ml) for 1 hour. Thereafter, 50 μl aliquots of medium were mixed with the same volume of a 40:1 ratio of Reagent A (H2O2 in Tris buffer) and Reagent B (acridan solution in dioxane and ethanol) in an Amersham ECL Plus kit (GE Healthcare), and then incubated for 5 minutes at RT with light protection. Luminescence intensity at an emission wavelength of 430 nm was measured using a microplate reader (SpectraMax Gemini XPS; Molecular Devices). Intracellular ROS were measured using 2',7'-dichlorodihydrofluorescein diacetate (DCFDA) dye, according to the manufacturer's instructions. After incubating for 24 hours in clear-bottom, 96-well black plates, RAW 264.7 cells were incubated with 20 μM DCFDA dye for 45 minutes at 3 °C in the dark and then washed gently with medium. Thereafter, PBS or 5 μg/ml LPS, with or without each SPION (Fe, 1 mM), was added, and fluorescence intensity was measured at excitation and emission wavelengths of 485 and 535 nm, respectively, using a fluorescence microplate reader (SpectraMax Gemini XPS; Molecular Devices).

### Gene expression analysis

Total RNA was harvested from RAW 264.7 cells using a Hybrid-R RNA purification kit (GeneAll Biotechnology, Seoul, Korea), according to the manufacturer's guidelines. After the extraction process, the concentration and purity of the isolated RNA were measured at 260 and 280 nm by spectrometry. cDNA was synthesized from total RNA (1 μg) by reverse transcription using the ImProm-II Reverse Transcription System (Promega Corp., Madison, WI, USA), according to the manufacturer's guidelines. The levels of target mRNAs were measured by quantitative RT-PCR in reaction mixtures containing 2 μl cDNA and primer pairs using a KAPA SYBR Fast qPCR kit (Kapa Biosystems Inc., Wilmington, MA, USA). Glyceraldehyde 3-phosphate dehydrogenase (GAPDH) mRNA was used as a housekeeping gene. The primer pairs used in this study are as follows: gp91phox (NOX2), 5'-CCA ACT GGG ATA ACG AGT TCA-3' (forward) and 5'-GAG AGT TTC AGC CAA GGC TTC-3' (reverse); and manganese-dependent superoxide dismutase (MnSOD), 5'-AAC TCA GGT CGC TCT TCA GC-3' (forward) and 5'-GCT TGA TAG CCT CCA GCA AC-3' (reverse). Relative gene expression was quantified using the ΔΔCt method, and the levels of expression of target genes were normalized against GAPDH expression and reported as fold changes relative to the control group.

### Prussian blue staining

Before Prussian blue staining, cells and abdominal wall tissues were fixed with 4% paraformaldehyde solution. An equal volume of a mixture of 2% potassium ferrocyanide (II) and 2% HCl was added and the solution was incubated for 20 minutes, then washed vigorously three times with distilled water. Thereafter, cells/tissues were counterstained with 1% neutral red for 5 minutes, followed by washing, dehydration, clearing, and mounting. Prussian blue staining results were assessed by light microscopy and images were adjusted using Image J.

### NMR measurement

NMR relaxometry was performed on a Bruker AVANCE 300 NMR spectrometer (Bruker Instruments, Billerica, MA, USA). Manual shimming was carried out before all measurements. T2 relaxation was measured using a standard Carr-Purcell-Meiboom-Gill (CPMG) pulse sequence 40. A total of 23,800 echoes were acquired with a TE of 20 ms. A radio-frequency excitation pulse with 90° flip angles and a duration of ~12.0 μs was used; two scans with at least triplicate measurements were performed for all experiments.

### NMR-based whole-blood assay and *in vivo* mouse sepsis model

For measurement and quantification of ROS (NaOCl) levels by NMR assay, blood samples were collected from the retro-orbital sinus of C57BL/6 mice without sacrificing. Blood was then incubated under predefined treatment conditions (each iron oxide nanoparticle ± stimuli) for various times, and then mixed with deuterated water (10%, v/v) to yield a final hematocrit of 10% before measurement. The samples thus prepared were placed in a 5 mm o.d. NMR sample tube for measurement. T2 relaxation times were obtained by processing and analyzing spectral data using MestReNOva 8.1.4 software (Umetrics, Umeå, Sweden). △T2 values for PEG-BR@SPIONs were determined by subtracting the T2 relaxation time obtained for PEG-DSPE@SPIONs (negative control). The basal T2 relaxation time for each iron oxide nanoparticle was evaluated prior to creating the sepsis model. After basal blood extraction, each mouse was given an intraperitoneal injection of LPS (100 μg/ml). Six hours later, infected blood was extracted through retro-orbital sampling and, after incubating for 12 hours with different SPION preparations under predefined conditions (see above), T2 relaxation time was measure. Rectal temperature, one surrogate marker of sepsis, was measured twice—once before LPS treatment and again 6 hours after LPS treatment. For the measurements of complete blood cell counts and differential white blood cell counts, the blood was drawn before euthanasia. Fluorescence images were acquired and analyzed using a Xenogen IVIS Lumina 100 imaging system (PerkinElmer, Waltham, MA, USA) or a FOBI.

### *In vivo* test of ROS responsiveness

Sepsis model mice (induced by injection of 100 μg/ml LPS) were anesthetized by intraperitoneal injection of a 30-mg/kg tiletamine/zolazepam solution containing 10 mg/ kg of xylazine, and then injected intraperitoneally with 200 μl of cypate-loaded iron oxide nanoparticles (Fe, ~1.5 mM). Six hours after treatment, mice were sacrificed, and *ex vivo* fluorescence images were acquired and analyzed using a Xenogen IVIS Lumina 100 imaging system (PerkinElmer, Waltham, MA, USA) or a FOBI fluorescence imaging system (NeoScience, Suwon, Korea) with an ICG filter channel.

### Isolation of peritoneal cells

Resident peritoneal cells were harvested according to the protocol of Zhang et al., with slight modifications 41. Briefly, the anesthetized mouse was placed in one hand and 5 ml of DMEM was slowly injected into the peritoneal cavity at the lower left quadrant of the shaved abdomen with a 5-ml syringe. Injection of media inflated the peritoneal cavity, providing sufficient separation between internal organs and the peritoneum. The mouse torso was then gently shaken several times to detach peritoneal cells from the tissue and transfer them into the medium. After gentle massage, the cell suspension was extracted from the peritoneal cavity with a 5-ml syringe. Extracted cell suspensions were kept on ice before assay.

### Flow cytometry analysis of cellular uptake

After extraction from the peritoneum, peritoneal cell suspensions were microcentrifuged for 10 minutes at 1,000 rpm (4°C), and cell pellets were resuspended in DMEM for cell counting. An equal number of peritoneal cells (1 × 10^6^) from each group was suspended in flow cytometry buffer (PBS containing 0.5% bovine serum albumin and 0.05% sodium azide), and then intracellular uptake of cypate was evaluated by flow cytometry using a LSR II flow cytometer (BD Biosciences, Franklin Lakes, NJ, USA). Cypate fluorescence intensity was measured by exciting at 650 nm and collecting emitted fluorescence using a 774 nm band-pass filter. Acquired data were analyzed using FlowJo software (Tree Star Inc., San Carlos, CA, USA).
