# Supplementary material for: PEGylated Bilirubin-coated Iron Oxide Nanoparticles as a Biosensor for Magnetic Relaxation Switching-based ROS Detection in Whole Blood
Source: Theranostics. 2020 Jan 12;10(5):1997–2007. doi: 10.7150/thno.39662 (PMC7019166; doi:10.7150/thno.39662)
Supplement: Supplementary file 1 — Supplementary figures. [file thnov10p1997s1.pdf]

Supplementary Information

PEGylated Bilirubin-coated Iron Oxide Nanoparticles as a Biosensor for Magnetic Relaxation Switching-based ROS Detection in Whole Blood

Dong Yun Lee,[a,b] Sukmo Kang,[c,d,e] Younghyun Lee,[c,d,e] Jin Yong Kim,[a,d,e] Dohyun Yoo,[c,d,e] Wonsik Jung,[c,d,e] Soyoung Lee,[c,d,e] Yong Yeon Jeong,[f] Kwangyeol Lee,[g] and Sangyong Jon[a,c,d,e,*]

[a]Graduate School of Medical Science and Engineering, Korea Advanced Institute of Science and Technology (KAIST), 291 Daehak-ro, Daejeon 34141, Republic of Korea

[b]Department of Nuclear Medicine, Asan Medical Center, University of Ulsan College of Medicine, 88, OLYMPIC-RO 43-GIL, Seoul 05505, Republic of Korea

[c]Department of Biological Sciences, KAIST, 291 Daehak-ro, Daejeon 34141, Republic of Korea

[d]KAIST Institute for BioCentury, KAIST, 291 Daehak-ro, Daejeon 34141, Republic of Korea

[e]Center for Precision Bio-Nanomedicine, KAIST, 291 Daehak-ro, Daejeon 34141, Republic of Korea

[f]Department of Radiology, Chonnam National University Hwasun Hospital, 322 Seoyang-ro, Hwasun 58128, Republic of Korea

[g]Department of Chemistry, Korea University, 145 Anam-ro, Seoul 02841, Republic of Korea

Corresponding Authors: Sangyong Jon. Phone: +82-10-47210602; Fax: +82-42-3504450; E-mail: syjon@kaist.ac.kr

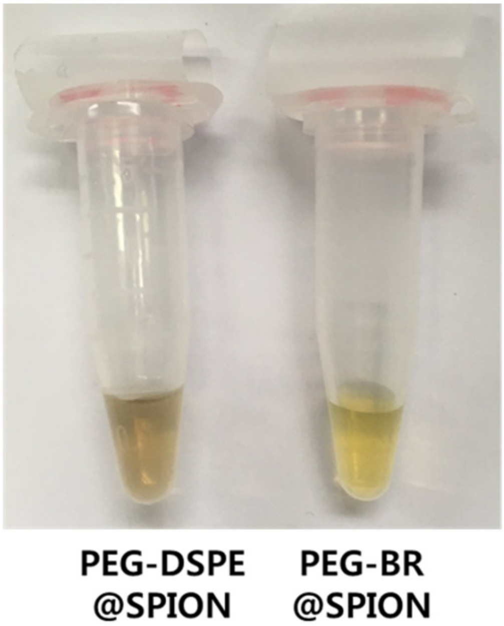

Figure S1. A photograph of as-prepared SPIONs dispersed in phosphate buffered saline (pH 7.4).

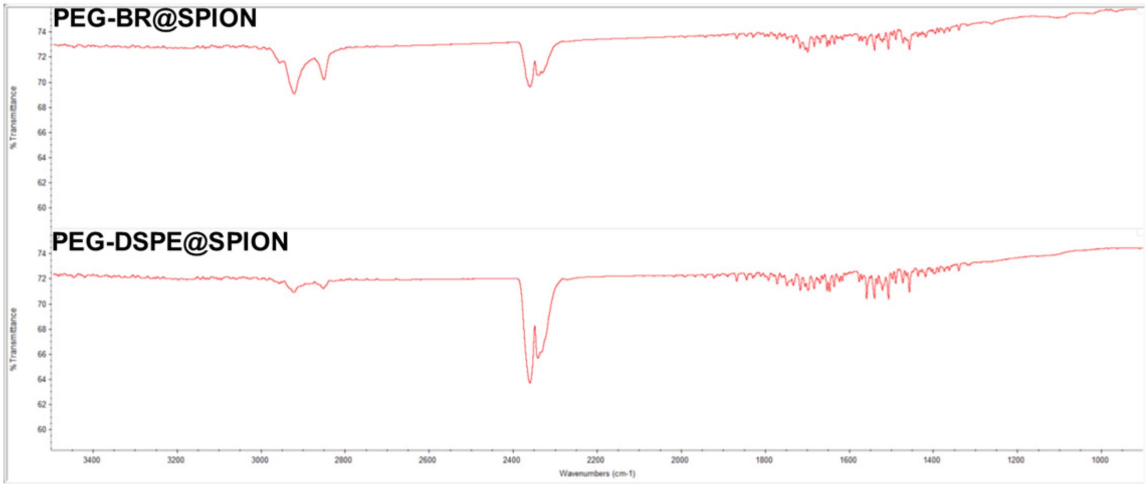

Figure S2. FT-IR spectra of as-prepared SPIONs.

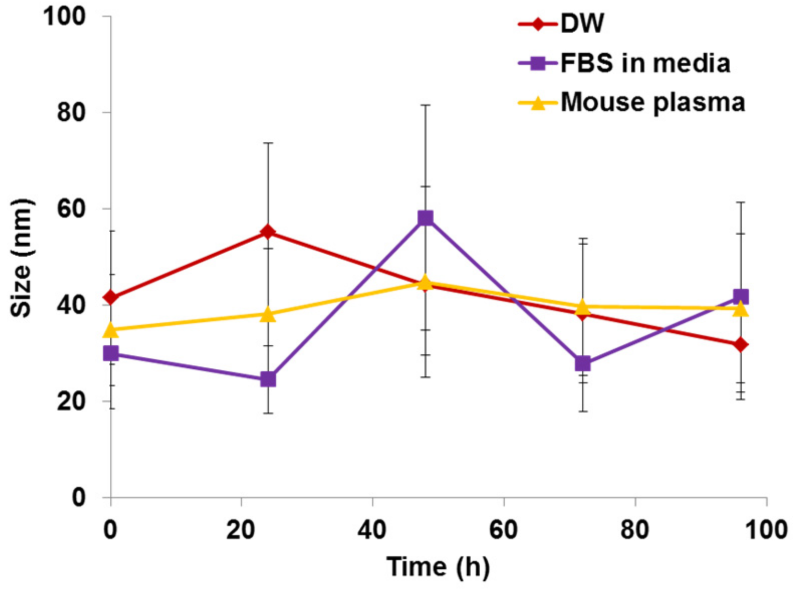

Figure S3. Hydrodynamic size measurements of PEG-BR@SPIONs under various conditions as a function of incubation time.

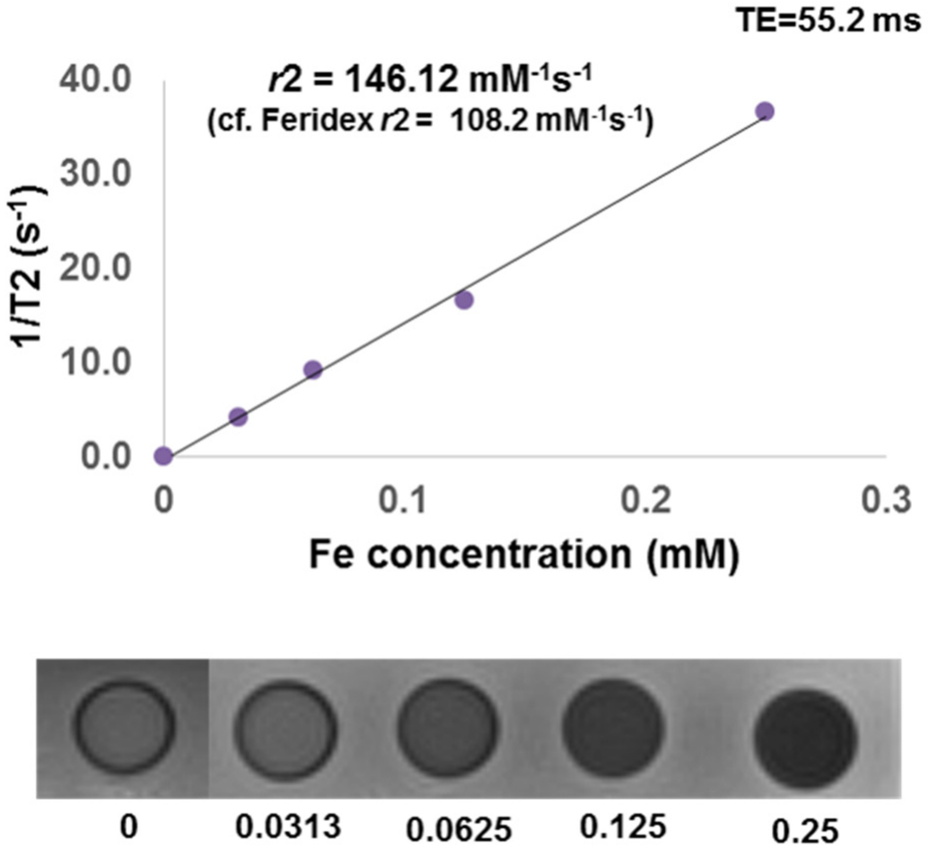

Figure S4. Relaxation rates of PEG-BR@SPIONs as a function of Fe concentrations and corresponding T_2 phantom images obtained using 3.0 T MRI.

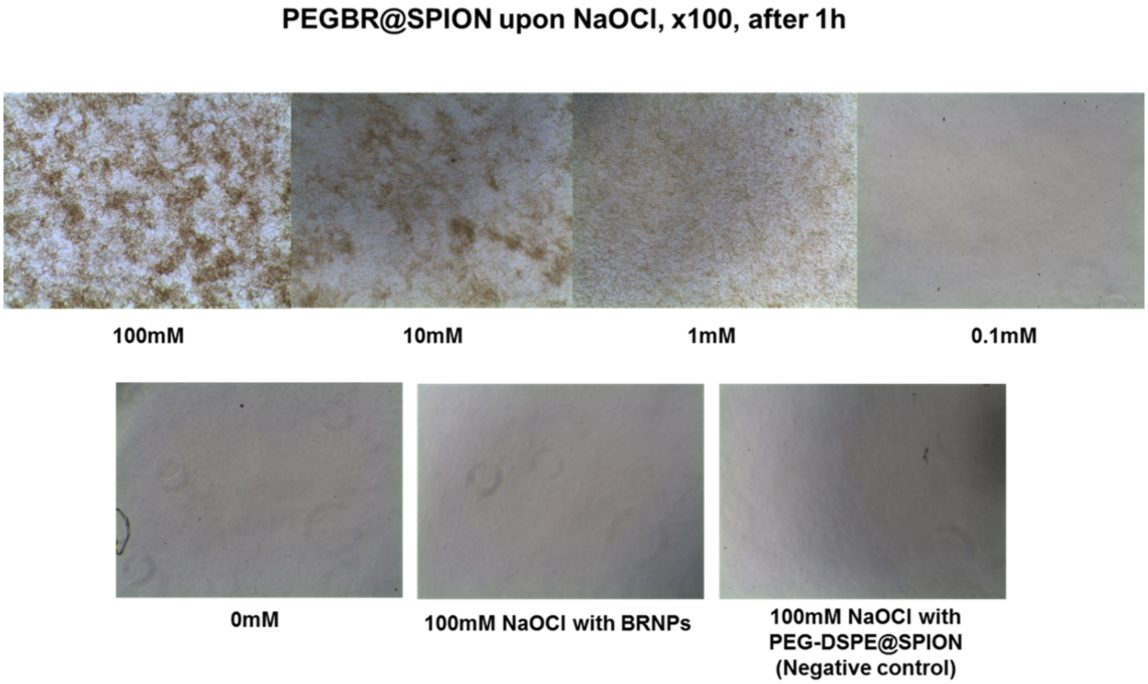

Figure S5. Light microscopic images of ROS concentration-dependent aggregation of PEG-BR@SPIONs after reacting with different concentrations of NaOCl. BRNPs denote nanoparticles derived from self-assembly of solely PEG-BR.

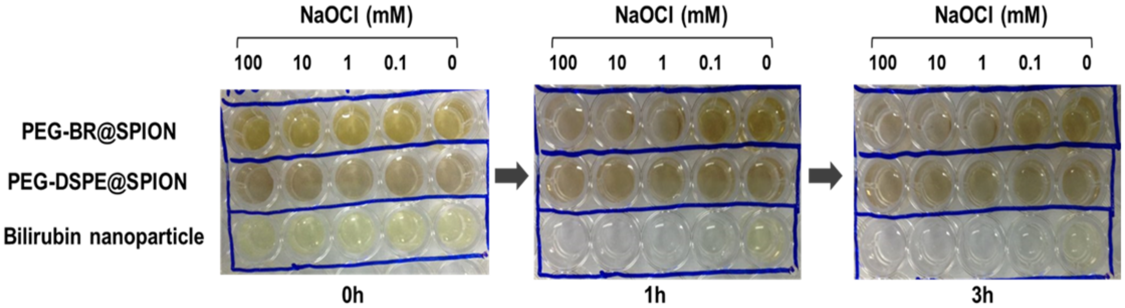

Figure S6. Kinetics of color changes of each nanoparticle solution upon incubation with different concentrations of NaOCl-derived ROS.

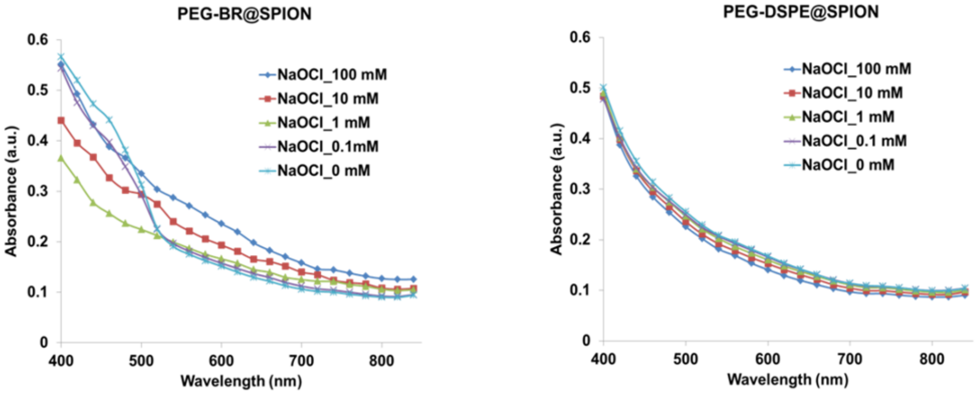

Figure S7. Comparison of absorbance changes between PEG-BR@SPIONs and PEG-DSPE@SPIONs after reacting with different concentrations of NaOCl-derived ROS. For PEG-BR@SPIONs, the change in the absorbance at 420 nm indicates that the chemical structure of BR is modified (fragmentized) upon reaction with ROS.

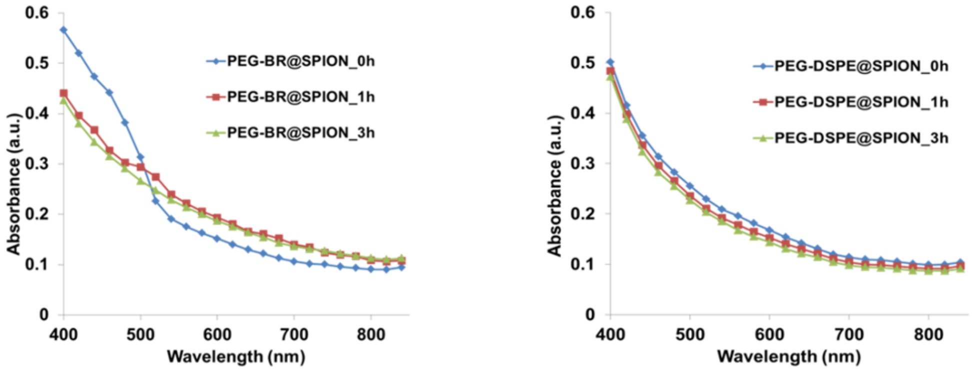

Figure S8. Comparison of the kinetics of absorbance changes between PEG-BR@SPIONs and PEG-DSPE@SPIONs after reacting with a fixed concentration of NaOCl-derived ROS (10 mM). For PEG-BR@SPIONs, the change in the absorbance at 420 nm indicates that the chemical structure of BR is modified (fragmentized) upon reaction with ROS.

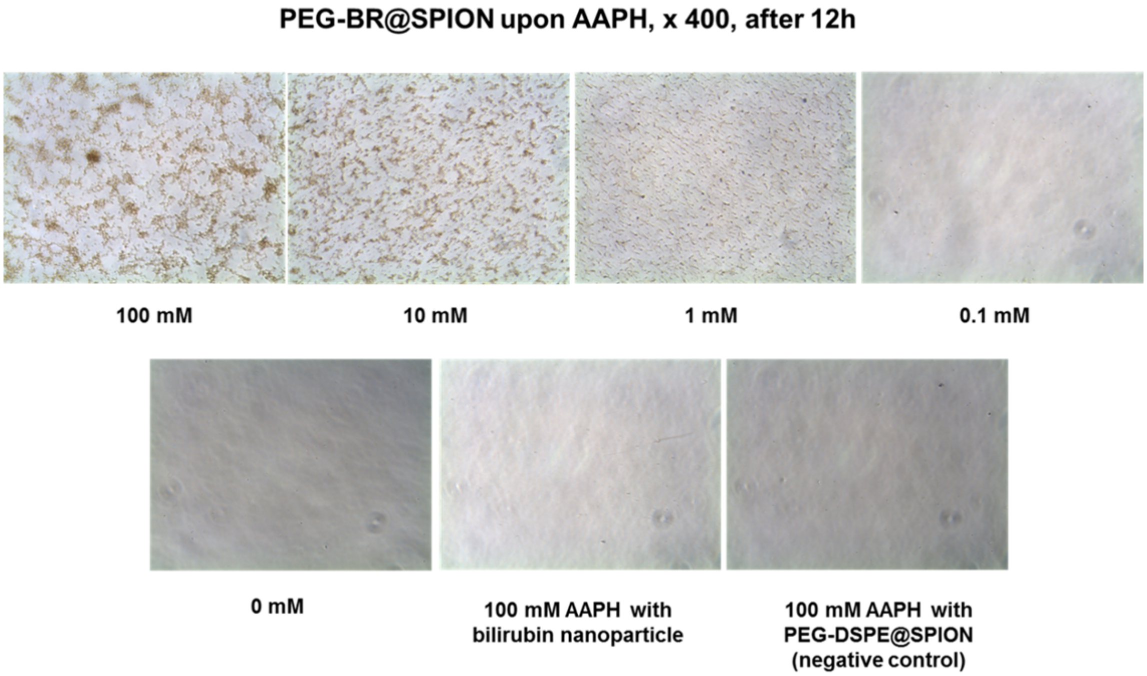

Figure S9. Light microscopic images of ROS concentration-dependent aggregation of PEG-BR@SPIONs after reacting with different concentrations of AAPH.

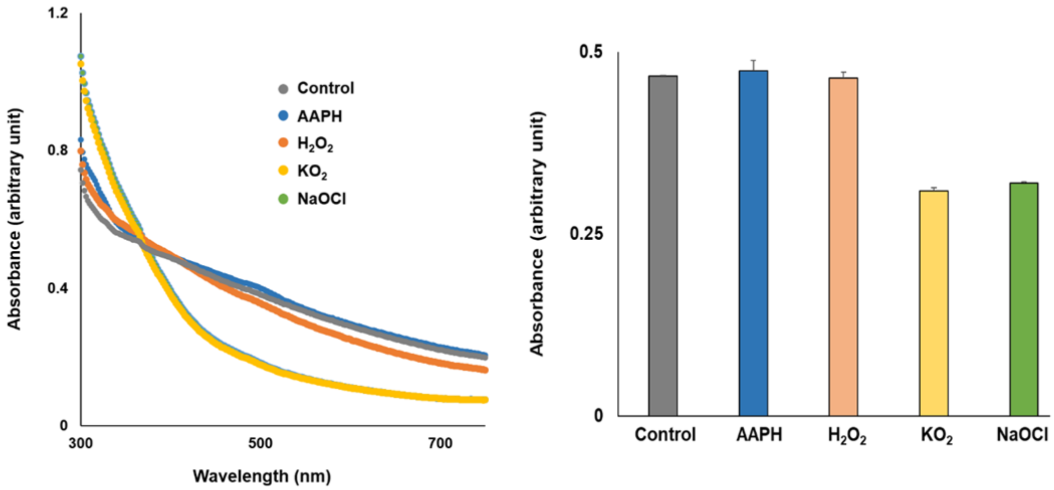

Figure S10. Left: UV-Vis Spectra of PEG-BR@SPION (Fe: 50 μM) taken after treatment with four different ROS (100 μM) for 12 hours. Right: Comparison of absorbance at 420 nm in the UV-Vis spectra on the left (n = 4).

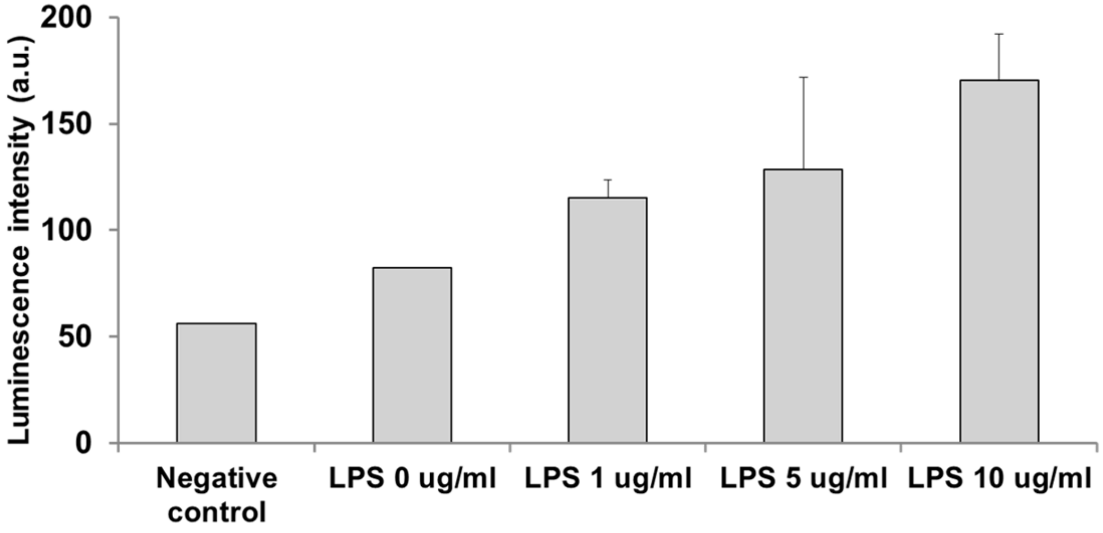

Figure S11. Measurements of extracellular ROS production in RAW 264.7 cells after treatments with increasing LPS concentrations.

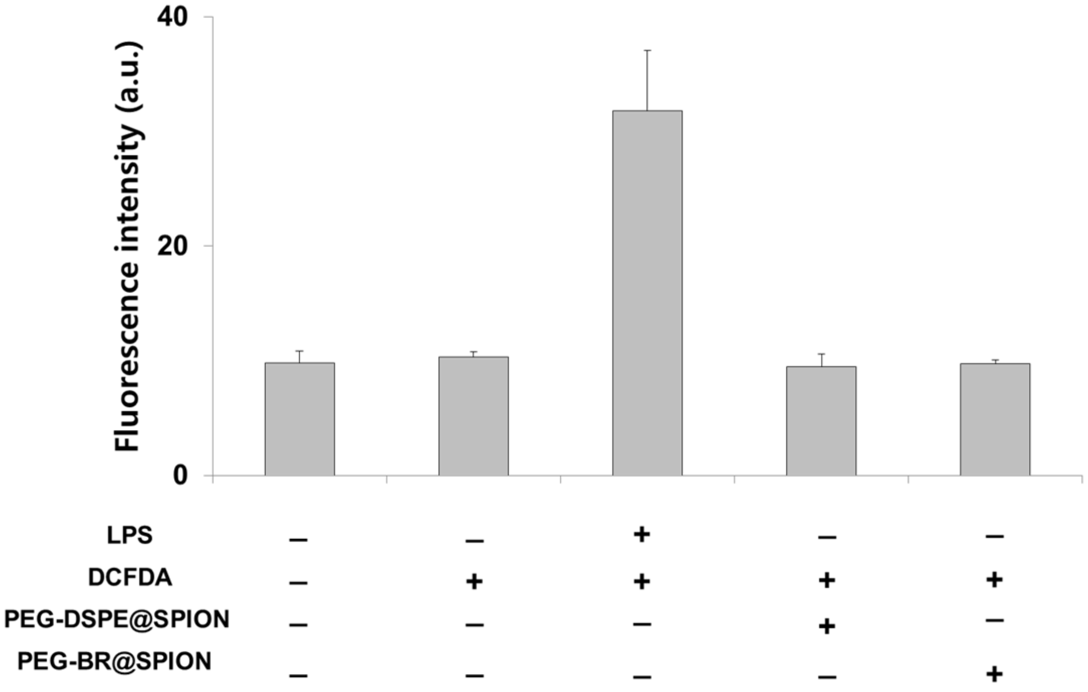

Figure S12. Measurements of intracellular ROS production in RAW 264.7 cells following various treatments.

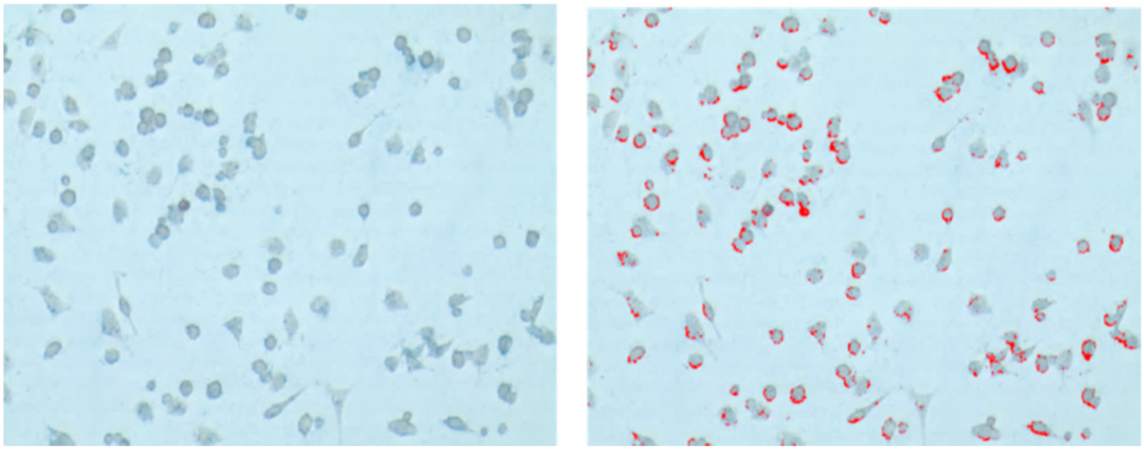

Figure S13. Prussian blue-stained (left) and corresponding pseudo-colored image (right) of RAW 264.7 cells after co-treatment with PEG-BR@SPIONs and LPS.

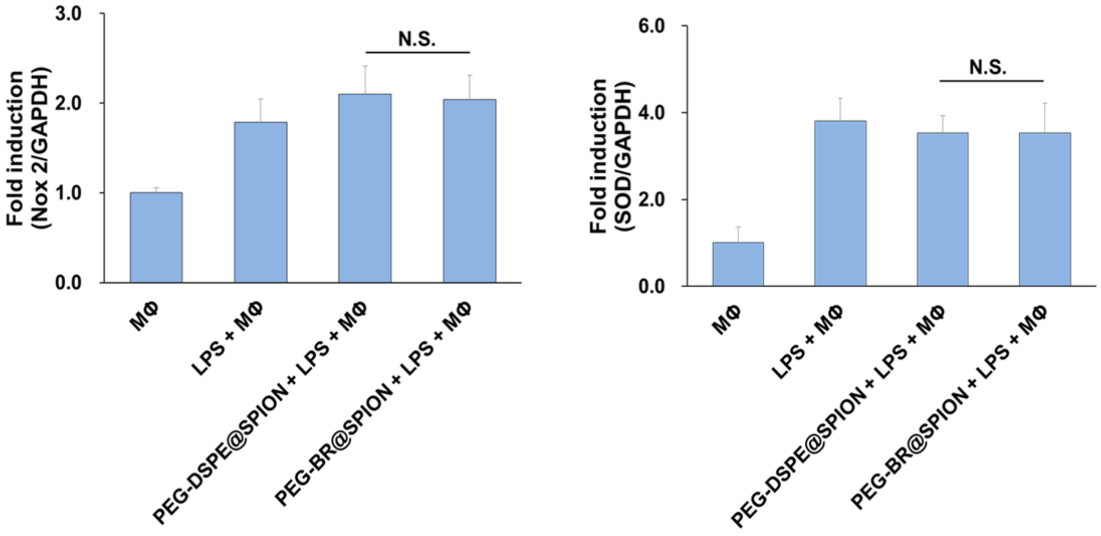

Figure S14. Relative mRNA expression levels of ROS-producing enzymes in RAW 264.7 cells in each treated group. Left: NADPH oxidase 2 (Nox2); right: superoxide dismutase (SOD).

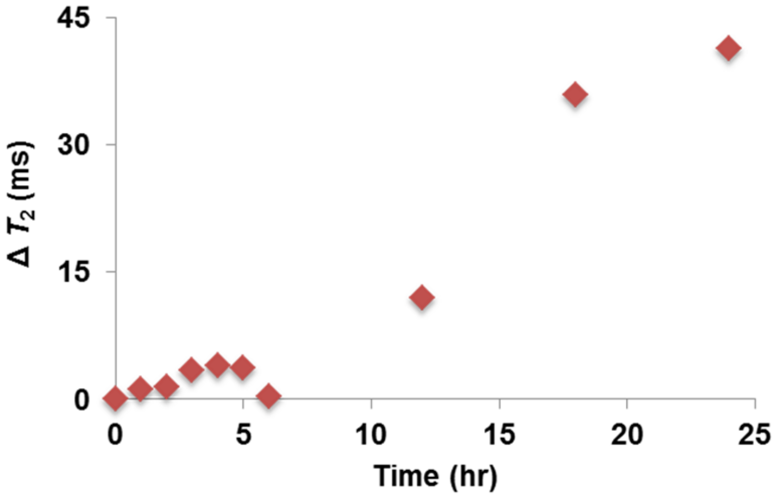

Figure S15. Kinetics of the ROS-sensing magnetic relaxation-switching assay of PEG-BR@SPIONs in whole blood after reacting with 300 μM NaOCl.

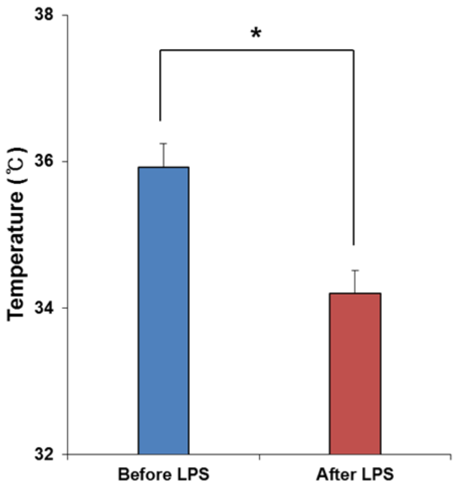

Figure S16. Body temperature of C57BL/6 mice measured before and 6 hours after intraperitoneal LPS injection.

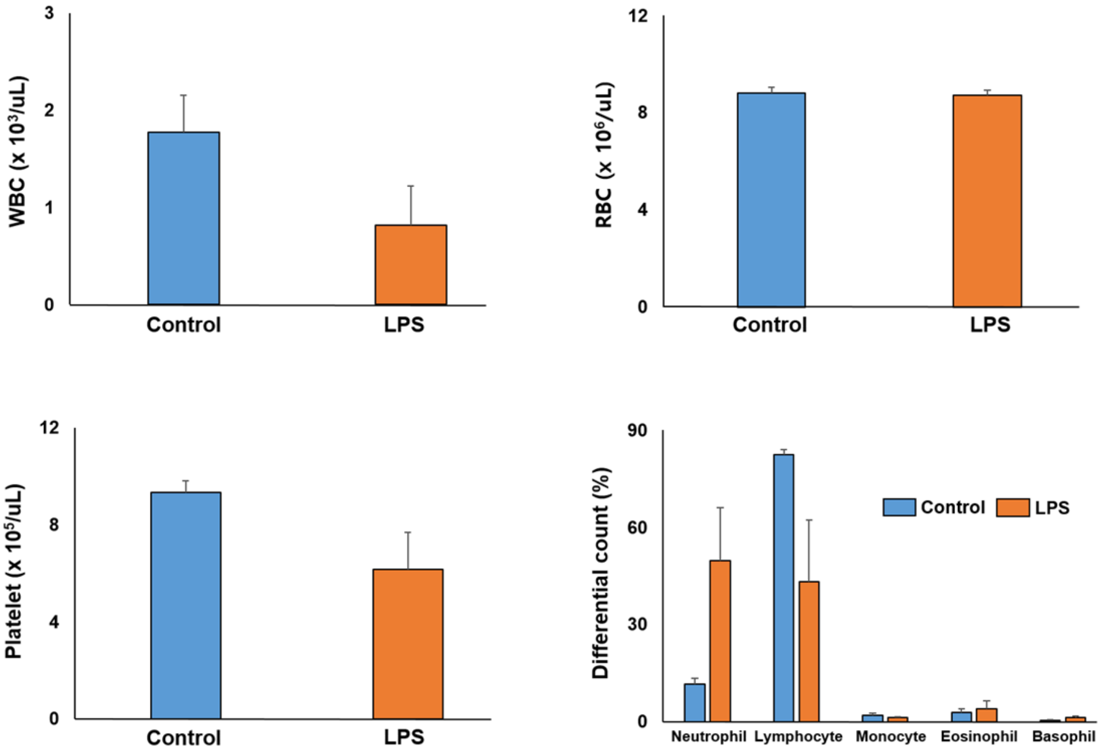

Figure S17. Complete blood cell counts and differential white blood cell (WBC) counts in C57BL/6 mice 6 hours after with/without intraperitoneal LPS injection (n = 3).

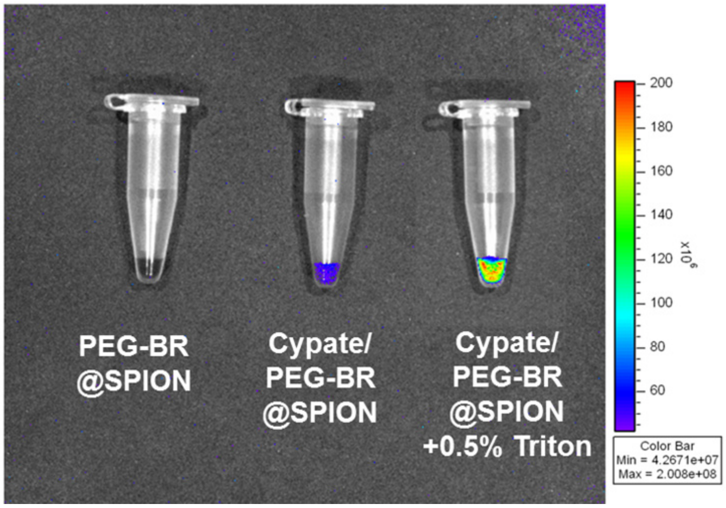

Figure S18. *In situ* fluorescence images of Cypate/PEG-BR@SPIONs before and after treatment with Triton X detergent.

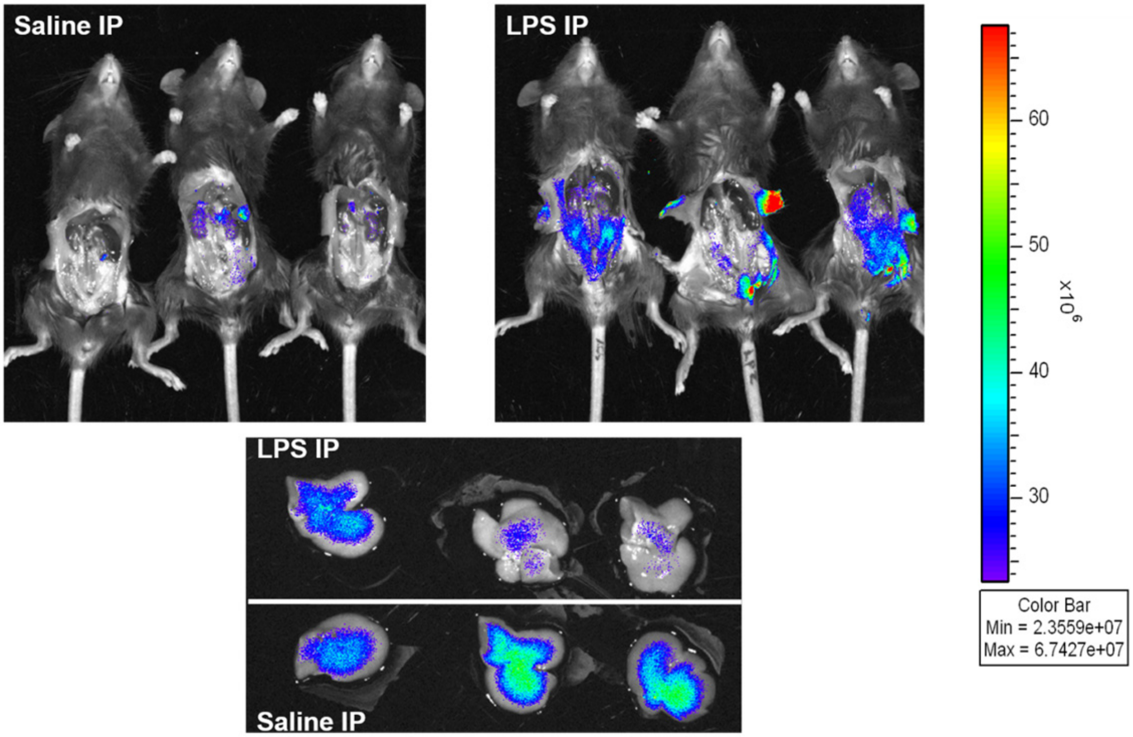

Figure S19. *Ex vivo* fluorescence images of Cypate/PEG-BR@SPIONs around the abdominal wall and in the liver after intraperitoneal injection of either saline or LPS (n = 3). The result obtained from the same experiment is shown in Figure 5c.

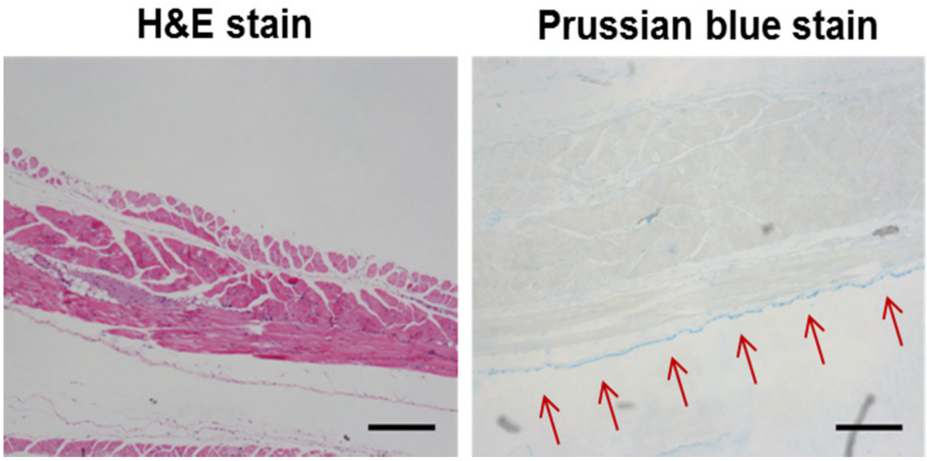

Figure S20. H&E-stained and corresponding Prussian blue-stained images of the abdominal wall of mice being treated with Cypate/PEG-BR@SPIONs and LPS presented in Figure 5C. Red arrows indicate the retained iron oxide nanoparticles along the parietal peritoneum. Scale bar, 100 µm.

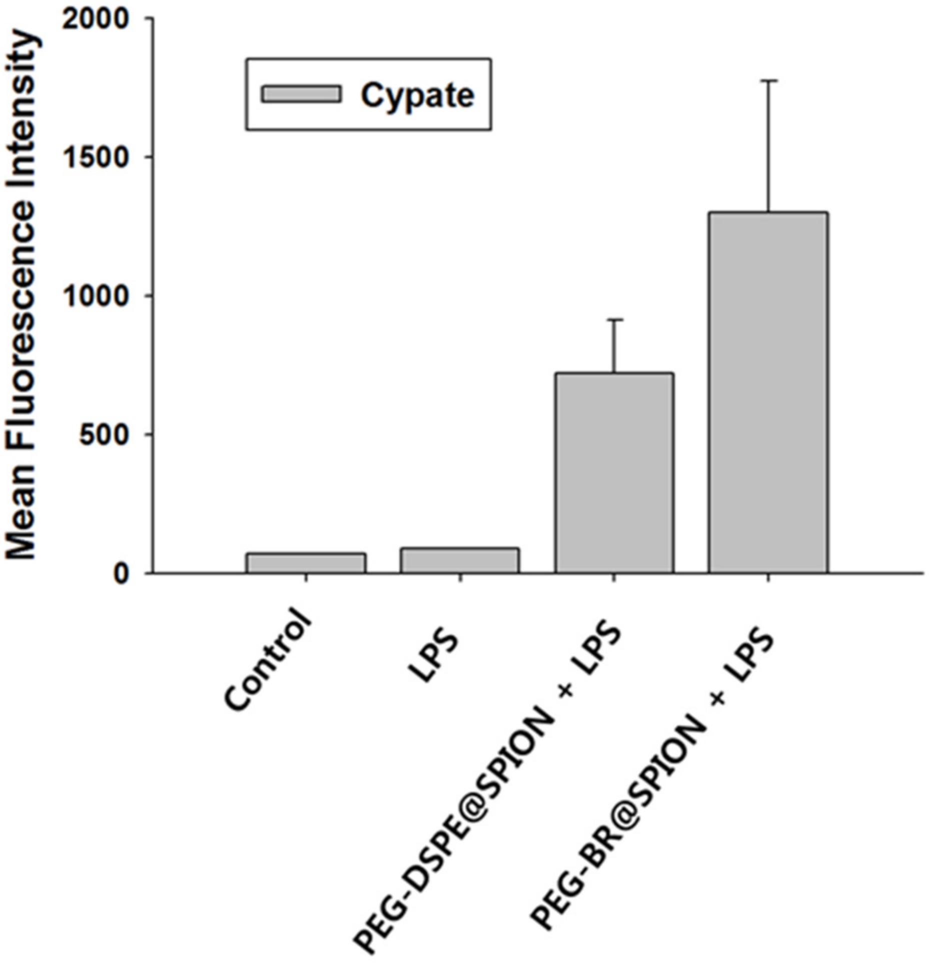

Figure S21. Mean fluorescence intensity of cypate uptake in the resident periton eal cells (n = 3). This result corresponds to Figure 5d.
